# Hyperactivity, perseveration and increased responding during attentional rule acquisition in the Fragile X mouse model

**DOI:** 10.3389/fnbeh.2013.00172

**Published:** 2013-11-21

**Authors:** Ioannis Kramvis, Huibert D. Mansvelder, Maarten Loos, Rhiannon Meredith

**Affiliations:** ^1^Department of Integrative Neurophysiology, Centre for Neurogenomics and Cognitive Research, VU University AmsterdamAmsterdam, Netherlands; ^2^Sylics (Synaptologics BV)Amsterdam, Netherlands; ^3^Department of Molecular and Cellular Neurobiology, Centre for Neurogenomics and Cognitive Research, VU University AmsterdamAmsterdam, Netherlands

**Keywords:** Fragile X, attention, hyperactivity, 5-choice serial reaction time task, learning, perseveration, MPEP, prefrontal cortex

## Abstract

Attentional deficits and executive function impairments are common to many neurodevelopmental disorders of intellectual disability and autism, including Fragile X syndrome (FXS). In the knockout mouse model for FXS, significant changes in synaptic plasticity and connectivity are found in the prefrontal cortex (PFC)—a prominent region for attentional processing and executive control. Given these alterations in PFC synaptic function, we tested whether adult Fragile X knockout mice exhibited corresponding impairments in inhibitory control, perseveration, and sustained attention. Furthermore, we investigated individual performance during attentional rule acquisition. Using the 5-choice serial reaction time task, our results show no impairments in inhibitory control and sustained attention. Fragile X knockout mice exhibited enhanced levels of correct and incorrect responding, as well as perseveration of responding during initial phases of rule acquisition, that normalized with training. For both knockout and wild type mice, pharmacological attenuation of metabotropic glutamate receptor 5 signaling did not affect response accuracy but reduced impulsive responses and increased omission errors. Upon rule reversal, Fragile X knockout mice made more correct and incorrect responses, similar to the initial phases of rule acquisition. Analogous to heightened activity upon novel rule acquisition, Fragile X knockout mice were transiently hyperactive in both a novel open field (OF) arena and novel home cage. Hyperactivity ceased with familiarization to the environment. Our findings demonstrate normal inhibitory control and sustained attention but heightened perseveration, responding, and hyperactivity during novel rule acquisition and during exposure to novel environments in Fragile X knockout mice. We therefore provide evidence for subtle but significant differences in the processing of novel stimuli in the mouse model for the FXS.

## Introduction

Prominent impairments in attentional processing and inhibitory control occur in many intellectual disability syndromes and autism spectrum disorders (ASD) (Hagerman, 2006; Scerif and Steele, 2011). In Fragile X syndrome (FXS), deficits in sustained and selective attention, impaired executive control and behavioral inflexibility are reported in both children and adults (Munir et al., 2000; Cornish et al., 2001; Scerif et al., 2007; Hooper et al., 2008). The prefrontal cortex (PFC) is prominently involved in attentional processing, executive function, inhibitory control, and rule acquisition in operant tasks (Dalley et al., 2004; Peyrache et al., 2009; Rossi et al., 2009). In the FXS mouse model (*Fmr1*-KO), significant alterations in synaptic connectivity and plasticity occur in PFC (Meredith et al., 2007; Gocel and Larson, 2012; Testa-Silva et al., 2012; Paul et al., 2013). Previous studies with adult *Fmr1*-KO mice report no deficits in sustained attentional performance compared to wildtype (WT) littermates (Krueger et al., 2011) while earlier findings indicated impairments in inhibitory control and in attentional processing (Moon et al., 2006). Given the disruption of multiple attentional components in FXS (Wilding et al., 2002; Scerif and Steele, 2011), and the role of PFC in specific executive functions and rule acquisition, we determined to assess inhibitory control, sustained attention and rule acquisition in the *Fmr1*-KO mouse.

Attentional processing can be subdivided into different task-specific components (Knudsen, 2007). In rodents, the five choice serial reaction time task (5CSRTT) is designed to test various aspects of attentional control, including sustained, selective and divided attention (Robbins, 2002). We challenged the mice on a visuo-spatial sustained-attentional paradigm, for their ability to maintain a consistent behavioral response during continuous repetitive activity. The accuracy with which mice performed this task was taken as a measurement of their attentional capacity and function. Refraining from prematurely responding to a food-predictive stimulus was used as an index of inhibitory control. Besides measuring attentional performance and inhibitory control in *Fmr1*-KO and WT mice under standard task conditions, we tested the effect of a metabotropic glutamate receptor 5 (mGluR5) inverse agonist 6-Methyl-2-(phenylethyny)pyridine (MPEP), a therapeutic candidate for FXS, upon performance in this attentional paradigm. Lastly, we investigated the degree of behavioral flexibility of *Fmr1*-KO mice when adapting to a rule reversal of the sustained attention task.

Hyperactivity is common to several monogenic models for neurodevelopmental disorders (Crawley, 2007) and the degree of activity can depend on the context in which it is measured (i.e., novel, familiar, anxiogenic). Thus, measuring activity in different contexts could provide a better understanding of the factors contributing to differences in activity between *Fmr1*-KO and WT mice (e.g., differences in emotionality, response to novelty, or general activity). To better understand activity differences between *Fmr1*-KO and WT mice, we investigated locomotion in a novel brightly-lit open field (OF) (putatively anxiogenic) at developmental stages both before and after behavioral testing, as well as in a novel and familiar home cage environment.

Our results demonstrate that *Fmr1*-KO adult male mice exhibit no impairments in sustained attention or inhibitory control in the standard 5CSRTT. KO mice were hyperactive in novel environments at developmental stages both before and after behavioral training and displayed enhanced rates of responding during acquisition of novel rules in the learning phases of the 5CSRTT. Heightened perseveration and increased responding in KO mice was significantly reduced during attentional training, as was hyperactivity upon familiarization with the environment. Furthermore, in both *Fmr1*-KO and WT mice, MPEP did not affect response accuracy but significantly attenuated impulsive responses and increased errors of omission. Finally, similar to learning phases of 5CSRTT, KO mice exhibited enhanced responding and significantly higher error rates following attentional rule reversal.

## Materials and methods

### Animals

To obtain male *Fmr1*-KO (Bakker et al., 1994) and male WT age-matched littermates we crossed heterozygote *Fmr1* C57BL/6J females with WT C57BL/6J males. Breeding females had been previously backcrossed more than 10 generations on the C57BL/6J line (Charles River). Upon weaning at 3 weeks postnatal, mice were separated by gender and socially-housed until 8–9 weeks postnatal age. From that point onward male mice used for experiments were housed in individual cages, with water and food *ad libitum* except during the 5CSRTT (7:00/19:00 lights on/off). Experiments were carried out in accordance with the European Communities Council Directive of 24 November 1986 (86/609/EEC), and with approval of the local animal care and use committee of the VU University.

### Open-field activity

OF activity was tested for two consecutive days in naïve, 10 week old mice (*n* = 20 KO, *n* = 18 WT) and in a cohort of 25 week old mice that had been previously tested in the 5CSRTT (*n* = 15 KO, *n* = 16 WT). Mice were placed at a corner of the white OF chamber (50l × 50 w × 35 h cm) directly below a single white fluorescent light bulb (130 lx), and their activity was recorded for 10 min (Viewer^2^, Biobserve, St. Augustin, Germany). For the analysis of exploration the chamber was virtually divided in 9 equal squares and entries into the center square, time spent in center, distance covered in the center, velocity through the center, total distance covered, the percentage of inactivity at 0.1 cm/s threshold, and the velocity during mobility were measured (Viewer^2^, Biobserve, St. Augustin, Germany). The experiment was performed during the subjects' light cycle. The chamber was wiped with 70% Ethanol between testing each subject.

### Novel home-cage activity

At 9 weeks of age, 1 week after initial single housing in conventional cages, a separate batch of mice (*n* = 23 KO, *n* = 25 WT) was placed in an automated home-cage (PhenoTyper model 3000, Noldus Information Technology, Wageningen, The Netherlands; 30 l × 30 w × 35 h cm) in the second half of the subjective light phase (14:00–16:00 h). The top unit of each cage contained an array of infrared LEDs and an infrared-sensitive video camera used for video-tracking. The X-Y coordinates of the mice' center of gravity, sampled at a resolution of 15 coordinates per second, were acquired and smoothed using EthoVision software (EthoVision HTP 2.1.2.0, based on EthoVision XT 4.1, Noldus Information Technology, Wageningen, The Netherlands). Data processing to generate the total distance moved was performed with the AHCODA™ analysis software (Synaptologics BV, Amsterdam, The Netherlands) as previously described in detail (Maroteaux et al., 2012).

### Pharmacology

The mGluR5 receptor inverse agonist 6-Methyl-2-(phenylethyny) pyridine (MPEP) (Sigma-Aldrich, St. Louis, MO, USA) was dissolved in physiological saline (0.9% sodium chloride) at 5 mg/mL stock concentration. Two different concentrations of MPEP were tested, 5 mg/kg (low), 20 mg/kg (high), in addition to saline control injection matching the volume of the highest MPEP dose. A 10 ml/kg maximum injection volume was used. The order of administration for each subject was randomly assigned and followed a within-subjects Latin square design. All injections were administered intraperitoneally (IP) 25 min prior to testing in the 5CSRTT. Pharmacology testing occurred every other day with a washout 5CSRTT session in between to avoid possible carry-over effects between different dosages.

### Five-choice serial reaction time task (5CSRTT)

The procedure was adapted from a previously published methodology (Loos et al., 2009). All 5CSRTT experiments were performed from 10:00–13:00, 5 days a week, during the subjects' light cycle. 15 *Fmr1*-KO and 16 WT age-matched littermates were tested. A week before the onset of the 5CSRTT, subjects were placed on a restricted diet and were gradually brought to 90% of their free feeding body weight. For the duration of the experiment the subjects were weighed daily and the amount of food provided was adjusted to maintain their body weight to 90%. Operant chambers were equipped with five response holes, a food magazine at the opposite wall and a house light. Both response holes and food magazine contained yellow LED stimulus lights and infrared response detectors. Each operant chamber was placed within sound attenuating ventilated cubicles.

On week 10, mice were introduced in the operant chamber for a single 20-min habituation session. Subsequently, mice performed two reward retrieval sessions during which food rewards (Dustless Precision Pellets, 14 mg, Bio-Serve, Frenchtown, NJ, USA) were delivered in the magazine at random fixed inter-trial intervals (ITI; 4, 8, 16, 32 s). Reward delivery coincided with switching on the magazine stimulus light, and ITI was initiated when the previous pellet has been collected during which the magazine stimulus light went off. A session ended after 25 min or upon initiating 50 trials.

Following magazine training, subjects entered Learning Phase 1 (L1) during which a trial started with all 5 response-hole lights illuminated. A poke in any of the response holes switched off all the 5 lights, switched on the stimulus light in the magazine, and delivered a reward into the magazine. Upon pellet collection the magazine stimulus light went off and an ITI (5 s) was initiated. A session lasted for 25 min or until 60 pellets were earned. The reaction time to any of the lit response holes after an ITI and the number of trials initiated were recorded. As soon as 50 or more pellets were collected or after 7 sessions, mice graduated to the next learning phase.

In Learning Phase 2 (L2) trials started with only one response-hole light illuminated. Responses in non-lit holes were of no consequence. A poke in the lit response-hole switched off the light, switched on the stimulus light in the magazine, and delivered a reward. Upon pellet collection the magazine stimulus light went off and an ITI (5 s) was initiated. Sessions lasted for 25 min or until 60 earned pellets. The reaction time to the lit response hole (correct reaction time), the number of pokes in the non-lit response-holes (incorrect pokes), and the number of trials initiated were recorded. As soon as 50 or more pellets were collected within one session or after a total of 7 sessions, mice graduated to the training phase.

During the training phase, a trial started with a response of the subject into the illuminated magazine, which switched off the magazine light and after an ITI of 5 s a response-hole stimulus light turned on for a limited duration (stimulus duration; SD). A poke in the correct response-hole during stimulus duration up to an additional limited hold (LH) of 4 s after the light went off, switched on the magazine stimulus light and delivered a reward in the magazine. An incorrect response to a non-lit response-hole or an omission of a response resulted in a 5 s time-out period during which all stimulus lights and house light were turned off. After the time-out both the house light and magazine stimulus light went on and the subject could initiate a new trial by poking into the magazine. A premature response into a non-illuminated hole during the ITI also resulted in a time-out period. In the first phase of the training stage SD was set to 16 s, and was gradually decreased to 8, 4, 2, 1.5 when the subject reached criterion (>30 Trials + >60% Accuracy + <60% Omissions) or after 7 consecutive sessions at any given SD stage. Errors of omission were defined as [100 × (omissions)/(omissions + correct responses + incorrect responses)]. Accuracy was defined as [100 × (correct responses)/(correct responses + incorrect responses)]. In a similar manner, accuracy was determined during the 1st half and 2nd half of session duration. Premature responses (impulsivity) were defined as [100 × (premature pokes)/(premature pokes + correct responses + incorrect responses)]. Perseverative responses in a lit response-hole were without consequences, but recorded as a measure of perseveration. Finally, latency to retrieve a pellet from the magazine as well as reaction times for a correct, incorrect, and premature response were also recorded. Sessions lasted for either 25 min or until 60 trials were reached. For all phases of the training stage the final session for each subject was used for analysis.

During the testing phase stimulus duration was decreased to 1 s and subjects were given 10 sessions. Experimental procedures and definitions were the same as the training stage. Baseline performance for each subject was calculated from the 6th to the 10th session.

Following the first testing phase (SD1), performance was also tested under two different MPEP concentrations with saline control trials. All mice received a saline injection at the end of their training session 1 week prior to drug testing, in order to habituate them to IP injections.

Finally, upon completion of pharmacology, mice were subjected to 10 reversal sessions to assess their ability to acquire a new rule after prolonged training. Reversal was similar to L2, however, a trial consisted of 4 response-hole stimulus lights on and 1 response-hole stimulus light off. A correct trial was scored as a poke in the non-illuminated response-hole. Upon a correct trial all response-hole lights went off, the magazine stimulus light switched on and a reward was delivered in the magazine. All responses to the illuminated response-holes were of no consequence and were recorded as incorrect pokes. No time-out period was assigned for any action during this phase. Sessions lasted for either 25 min or until 60 trials were reached.

### Statistical analysis

Data were analyzed as indicated in the figure legends. Differences between groups were analyzed with a Student's *t*-test for data following a parametric distribution, and the Mann-Whitney test for non-parametric data. A Welch's *t*-test was used when parametric data exhibited unequal distribution of variances. For non-parametric paired test analysis the Wilcoxon matched pairs test was used. A two-way repeated measures ANOVA was used for the analysis of perseverative responses, correct and incorrect responses, sessions to criterion, trials initiated, reward latency, correct reaction time, for the effects of MPEP, and for correct, incorrect responses during the reversal paradigm; a Bonferroni post-test analysis was used to compare the different means. Pearson or Spearman coefficients were used to analyse parametric and non-parametric correlations, respectively. Linear regression analysis was used to model the relationship between OF activity and open-field center entries. A two-way repeated measures ANOVA was used for the analysis of distance covered, mobility, and velocity in the novel OF arena and in the novel home cage; a Bonferroni post-test analysis was used to compare the different means. Analysis was performed with GraphPad Prism and IBM SPSS Statistics packages.

## Results

### Transient hyperactivity in response to novel environments in *Fmr1*-KO mice

**Figure 1:**
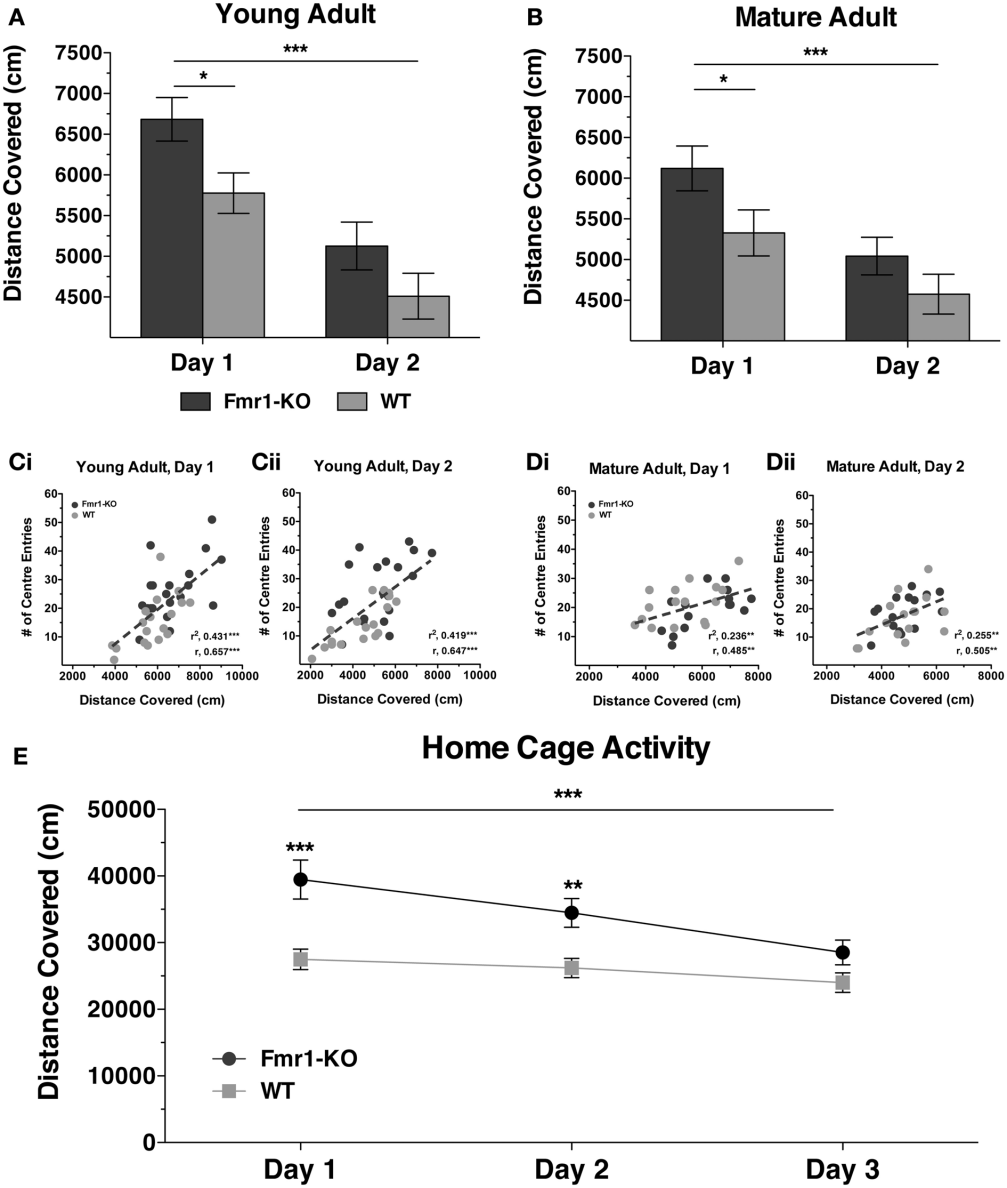
**Activity of young and mature adult *Fmr1*-KO mice in novel environments**. *Fmr1*-KO mice covered significantly greater distance than WT age-matched littermates during the first but not the second day of testing in a novel open field arena at 10 **(A)** and 25 **(B)** postnatal weeks; values plotted represent means ± SEM. The total distance covered correlated significantly with the number of center entry crossings for both 10 **(Ci**,**ii)** and 25 **(Di**,**ii)** week old mice during both days of testing; filled circles represent values from individual mice, dotted lines show linear regressions, r denotes Pearson correlation coefficient. Distance covered in novel home-cage was greater in young adult *Fmr1*-KO mice during the first two dark-cycles but not during the third dark-cycle **(E)**. For panels **(A), (B)** and **(E)** data were analyzed with a 2-way repeated measures ANOVA with Bonferroni's post-test analysis. For all panels asterisks indicate significance levels; ^***^p < 0.001, ^**^p < 0.01, ^*^p < 0.05.

Hyperactivity is a behavioral phenotype common to many different monogenic mouse models for neurodevelopmental disorders (Pliszka, 1998; Crawley, 2007) and can have a significant impact on learning abilities. To determine whether our *Fmr1*-KO mouse colony on a C57BL/6J background strain displayed a hyperactive phenotype, general activity of mice was tested in a conventional novel OF arena and in a novel home-cage (Figure 1). At 10 weeks age (young adult) *Fmr1*-KO mice covered significantly greater distances than WT littermates during the first but not the second day of testing in the novel OF arena (Figure 1A {2-way repeated measurements ANOVA [Days effect *F*_(1, 36)_ = 137.7, *p* < 0.0001], [Genotype effect *F*_(1, 36)_ = 4.23, *p* < 0.05], [Interaction effect *F*_(1, 36)_ = 1.46, *p* = 0.24]}). In a similar manner mature adult *Fmr1*-KO mice (25 weeks of age) exhibited hyperactivity that normalized during the second day in the novel OF arena (Figure 1B {2-way repeated measurements ANOVA [Days effect *F*_(1, 29)_ = 25.37, *p* < 0.0001], [Genotype effect *F*_(1, 29)_ = 4.20, *p* < 0.05], [Interaction effect *F*_(1, 29)_ = 0.63, *p* = 0.43]}). For both young and mature adult stages, increased velocity but not increased mobility was underlying the transient hyperactivity in *Fmr1*-KO mice (Supplementary Figure 1). Furthermore, the distance covered during both days of exploration significantly correlated with the number of “center entries” for both young (Figures 1Ci,ii, [Day1: (*r* = 0.657, *p* < 0.0001); Day2: (*r* = 0.647, *p* < 0.0001)]) and for mature adults (Figures 1Di,ii, [Day1: (*r* = 0.485, *p* < 0.01); Day2: (*r* = 0.505, *p* < 0.01)]). However, there was no significant difference between genotypes for overall time spent in the center suggesting no difference in anxiety-related behavior {Young Adult: Day1 [18.76 ± 1.95 KO, 15.13 ± 2.48 WT] *p* = 0.25; Day2 [15.18 ± 2.01 KO, 12.43 ± 2.14] *p* = 0.37; Mature Adult: Day1 [16.31 ± 2.19 KO, 18.75 ± 1.71 WT] *p* = 0.38, Day2 [15.86 ± 1.77 KO, 14.38 ± 2.51] *p* = 0.63 (in seconds)}. In addition to hyperactivity during a 10 min OF test, *Fmr1*-KO mice covered significantly greater distance during the first two dark cycles in a novel home-cage that normalized by the third cycle (Figure 1E {2-way repeated measurements ANOVA [Days effect *F*_(2, 92)_ = 17.96, *p* < 0.0001], [Genotype effect *F*_(1, 46)_ = 12.26, *p* = 0.001], [Interaction effect *F*_(2, 92)_ = 4.74, *p* < 0.01]}). Thus, *Fmr1*-KO mice exhibited hyperactivity in response to novel environments, which normalizes with repeated exposure and familiarization, in the absence of anxiety-related behavior.

### Enhanced responding by *Fmr1*-Ko mice during attentional rule acquisition in 5CSRTT

**Figure 2:**
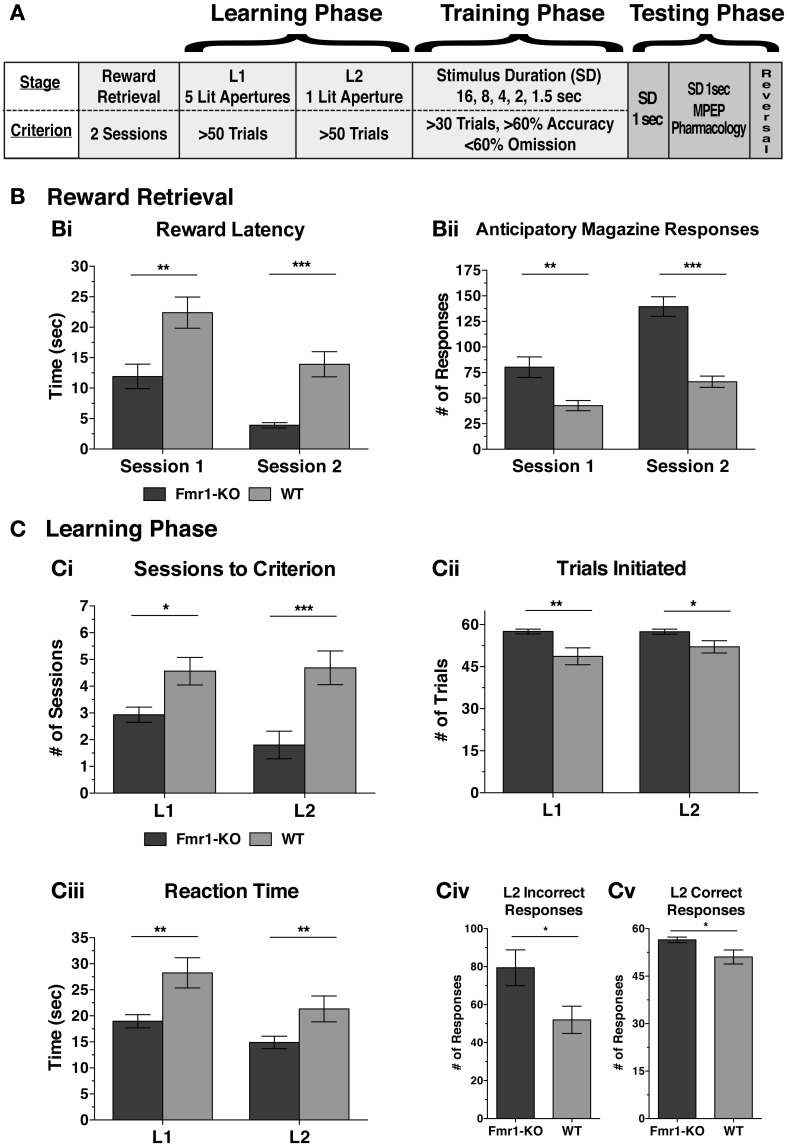
***Fmr1-KO mice progress faster during the activity-*dependent learning phase of the 5-CSRTT**. Overview of the sequence of learning, training, and testing phases during the 5CSRTT **(A)**. *Fmr1*-KO mice reacted more quickly to the illuminated magazine **(Bi)** with more anticipatory responses **(Bii)** during both reward retrieval sessions. *Fmr1*-KO mice achieved criterion in fewer sessions than WT during both learning phase 1 (L1) and 2 (L2) **(Ci)**, and initiated significantly more trials at both L1 and L2 **(Cii)**. *Fmr1*-KO mice reacted more quickly to the onset of a stimulus light presented in any of the five response holes (L1) and in one of the five (i.e., correct) response holes (L2) **(Ciii)**. During L2, *Fmr1*-KO mice committed significantly more incorrect **(Civ)** and correct **(Cv)** responses. Values plotted represent means ± SEM. The difference between groups was calculated using the Mann-Whitney test [**(Bi)**, **(Ci-L2)**, **(Cii)**, **(Ciii)**, **(Cv)**], *t*-test with Welch's correction of variance [**(Bii)**, **(Ci-L1)**] or *t*-test alone **(Civ)**. Asterisks indicate significance levels at; ^***^p < 0.001, ^**^p < 0.01, ^*^p < 0.05.

Executive function deficits are common in patients with neurodevelopmental disorders (Scerif and Steele, 2011). The 5CSRTT is an established methodology to test executive function such as attention, inhibitory control, and perseveration (Robbins, 2002). Prior to the training and testing phases of 5CSRTT, mice learned to associate the magazine with reward retrieval, and a response to an illuminated response-hole with a reward delivered in the magazine (Figure 2A). During both reward retrieval sessions *Fmr1*-KO mice responded significantly quicker to the illuminated magazine (Figure 2Bi {Session 1: [11.92 ± 2.01 KO, 22.40 ± 2.57 WT] *p* < 0.01; Session 2: [3.90 ± 0.43 KO, 13.92 ± 2.06 WT] *p* < 0.001}) and also with significantly more anticipatory responses (Figure 2Bii {Session 1: [80.27 ± 10.03 KO, 42.69 ± 5.00 for WT] *p* < 0.01; Session 2: [139.5 ± 10.0 KO, 66.06 ± 5.40 WT] *p* < 0.001}).

With the initiation of the learning phase, mice had to achieve a certain criterion in order to progress to the subsequent stage. During both learning phases 1 and 2 (L1, L2) *Fmr1*-KO mice progressed to the next phase with significantly fewer sessions than WT age-matched controls (Figure 2Ci) by initiating more trials at both L1 and L2 (Figure 2Cii {L1: [57.53 ± 0.86 KO, 48.69 ± 2.99 WT] *p* < 0.01; L2: [57.47 ± 0.88 KO, 52.06 ± 2.21 WT] *p* < 0.05}). This pattern was not due to motivational differences since the latency to retrieve a reward was equal between the two genotypes for both learning sessions {L1: [3.33 ± 0.27 KO, 3.85 ± 0.40 WT] *p* = 0.17; L2: [3.42 ± 0.64 KO, 3.27 ± 0.39 WT] *p* = 0.80 (in seconds)} and body weight restriction was equal between the two genotypes {L1: [87.63 ± 0.56 KO, 86.66 ± 0.47 WT] *p* = 0.20; L2: [89.51 ± 0.73 KO, 89.38 ± 0.43 WT] *p* = 0.88 (% free feeding body weight)}, as it was throughout the duration of the 5CSRTT (data not shown). Reaction time to any of the five illuminated response-holes in L1 or the only illuminated response-hole in L2 was significantly faster for *Fmr1*-KO mice (Figure 2Ciii {L1: [18.96 ± 1.26 KO, 28.26 ± 2.89 WT] *p* < 0.01; L2: [14.90 ± 1.19 KO, 21.34 ± 2.49 WT] *p* < 0.01}). Furthermore, for both groups, reaction time significantly correlated with the number of sessions to criterion {L1: *r* = 0.508, *p* = 0.003; L2: *r* = 0.364, *p* < 0.05}. Levels of poking in the incorrect non-illuminated response-holes during L2 were significantly higher for *Fmr1*-KO mice (Figure 2Civ [79.40 ± 9.43 KO, 52.00 ± 7.17 WT] *p* < 0.05), as was poking in the correct illuminated response-hole (Figure 2Cv [56.47 ± 3.42 KO, 51.00 ± 8.85 WT] *p* < 0.05). Therefore, *Fmr1*-KO mice responded significantly quicker during the 5CSRTT learning phase, initiated significantly more trials, committed both more correct and incorrect responses—all indicative of elevated activity levels during rule acquisition.

### Training normalizes *Fmr1*-KO performance and activity

With the initiation of the training phase both *Fmr1*-KO and WT, at the same rate, progressively required more sessions to reach criterion to commence to the next SD (Supplementary Figure 2A {2-way repeated measurements ANOVA; [Sessions effect *F*_(4, 116)_ = 10.09, *p* < 0.0001], [Genotype effect *F*_(1, 29)_ = 0.51, *p* = 0.48], [Interaction effect *F*_(4, 116)_ = 0.73, *p* = 0.57]}). Trials initiated by both groups remained constant throughout the training phase of the 5CSRTT (Supplementary Figure 2B {2-way repeated measurements ANOVA; [Sessions effect *F*_(5, 145)_ = 1.94, *p* = 0.11], [Genotype effect *F*_(1, 29)_ = 0.01, *p* = 0.89], [Interaction effect *F*_(5, 145)_ = 0.60, *p* = 0.70]}). With successive shortening of stimulus duration the reaction time to the correct response-aperture decreased significantly for both groups (Supplementary Figure 2C{2-way repeated measurements ANOVA; [Sessions effect *F*_(5, 145)_ = 219.8, *p* < 0.0001], [Genotype effect *F*_(1, 29)_ = 2.34, *p* = 0.14], [Interaction effect *F*_(5, 145)_ = 0.67, *p* = 0.64]}). Finally, motivation for the task did not change during the training phase for either group (Supplementary Figure 2D {2-way repeated measurements ANOVA; [Sessions effect *F*_(5, 145)_ = 1.77, *p* = 0.12], [Genotype effect *F*_(1, 29)_ = 0.12, *p* = 0.74], [Interaction effect *F*_(5, 145)_ = 0.47, *p* = 0.80]}). Therefore, during the training phase the activity and performance of *Fmr1*-KO mice normalized to that of WT age matched controls.

### Increased perseverative responding in *Fmr1*-KO mice at the onset of training

**Figure 3:**
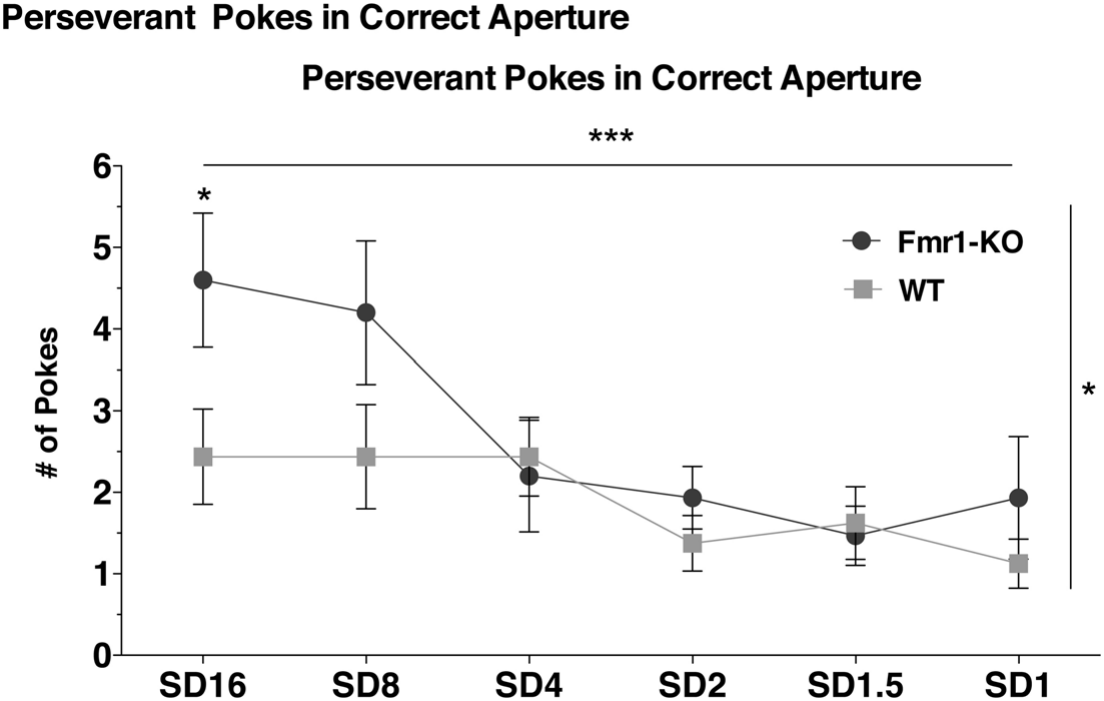
**Heightened perseverative behavior in *Fmr1*-KO mice**. *Fmr1*-KO and WT littermate mice significantly reduced the number of perseverative responses made in the correct illuminated hole over the entire training phase. However, *Fmr1*-KO mice poked significantly more at SD16 than WT. Data was analyzed with a 2-way repeated measures ANOVA with Bonferroni's post-test analysis. Asterisks indicate significance levels; ^***^p < 0.001, ^*^p < 0.05.

Perseveration, as deduced from the number of perseverative pokes in the correct response-hole, significantly decreased over the entire training phase and at the same rate for both genotypes (Figure 3 {2-way repeated measurements ANOVA [Session duration effect *F*_(5, 145)_ = 5.75, *p* < 0.0001]}, [Interaction effect *F*_(5, 145)_ = 1.82, *p* = 0.11]). However, perseverative poking was significantly higher for *Fmr1*-KO mice (Figure 3 {2-way repeated measurements ANOVA [Genotype effect *F*_(1, 29)_ = 4.33, *p* = 0.04]}). *Post-hoc* analysis showed that KO mice poked significantly more at the initial training session (SD16) compared to WT littermates (Figure 3, Bonferroni *p* < 0.05).

### Altered cross-trial performances in individual *Fmr1-KO mice*

**Figure 4:**
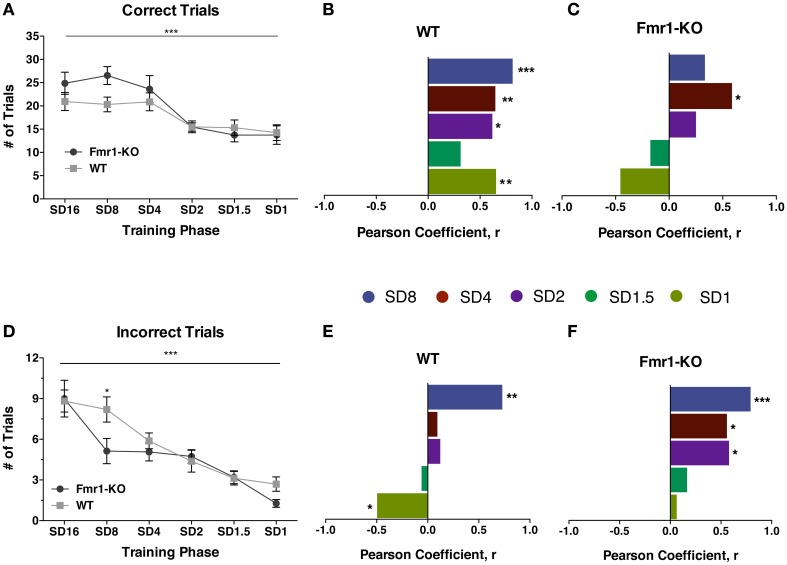
**Individual *Fmr1*-KO mice fail to sustain correct response performance and are delayed in inhibiting incorrect responses**. Correct trials are reduced for both *Fmr1*-KO and WT mice with progressively shorter stimulus duration during the 5CSRTT training and SD1 testing phase **(A)**. Individual WT mice exhibited a consistent correlation in correct performance between SD16 and subsequent stages **(B)**. *Fmr1*-KO individual mice failed to maintain a consistent correlation in correct performance **(C)**. Incorrect responses are significantly reduced for both *Fmr1*-KO and WT groups during the 5CSRTT successive stages **(D)**. No consistent correlation for incorrect performance for individual WT mice between SD16 and subsequent stages **(E)**. Individual *Fmr1*-KO mice showed consistent correlations for incorrect performance for most of the 5CSRTT stages **(F)**. For panels **(A)** and **(D)** data were analyzed with a 2-way repeated measures ANOVA with Bonferroni's post-test analysis. For all panels asterisks indicate significance levels; ^***^p < 0.001, ^**^p < 0.01, ^*^p < 0.05.

Progression through the training stage of the 5CSRTT became increasingly difficult, reflected by the significant reduction in the number of correct responses in both groups (Figure 4A {2-way repeated measurements ANOVA [Training phase effect *F*_(5, 145)_ = 16.73, *p* < 0.0001], [Genotype effect *F*_(1, 29)_ = 1.134, *p* = 0.30], [Interaction effect *F*_(5, 145)_ = 1.841, *p* = 0.109]}). Correlating the correct performance of individual mice at SD16 with all subsequent stages revealed that individual WT mice performed consistently from one training phase to the next (Figure 4B [Pearson correlation coefficient: SD8 0.814 (*p* = 0.0001), SD4 0.648 (*p* = 0.0067), SD2 0.618 (*p* = 0.014), SD1.5 0.313 (*p* = 0.237), SD1 0.6541 (*p* = 0.008)]). However, even though by the end of training *Fmr1*-KO mice as a group performed similarly to WT controls, KO individuals failed to sustain a consistent performance for correct responses through the training phases (Figure 4C [Pearson correlation coefficient: SD8 0.339 (*p* = 0.223), SD4 0.587 (*p* = 0.022), SD2 0.25 (*p* = 0.368), SD1.5 −0.175 (*p* = 0.533), SD1 −0.455 (*p* = 0.089)]). Whereas the best-performing WT mice with the highest number of correct responses at SD16 also had the highest number of correct responses at SD1, this pattern did not hold for *Fmr1*-KO mice: those with the highest number of correct responses at SD16 did not consistently exhibit the highest correct responses at SD1.

For both genotypes, training reduced the number of incorrect responses made during the entire training phase from SD16 to SD1 (Figure 4D {2-way repeated measurements ANOVA [Training phase effect *F*_(5, 145)_ = 27.15, *p* < 0.0001], [Genotype effect *F*_(1, 29)_ = 1.682, *p* = 0.205], [Interaction effect *F*_(5, 145)_ = 1.880, *p* = 0.101]}). Individual WT mice quickly learned to refrain from making incorrect responses, seen by the sharp drop in correlation between incorrect trials from SD16 to SD4 (Figure 4E [Pearson correlation coefficient: SD8 0.730 (*p* = 0.001), SD4 0.093 (*p* = 0.731), SD2 0.122 (*p* = 0.653), SD1.5 −0.068 (*p* = 0.802), SD1 −0.496 (*p* = 0.05)]). By SD1, individual WT mice initially making the most incorrect responses were making the least errors. In contrast, individual *Fmr1*-KO mice demonstrated a persistent correlation in the number of incorrect responses made for the majority of the training phase until SD1.5 (Figure 4F [Pearson correlation coefficient: SD8 0.791 (*p* < 0.0001), SD4 0.558 (*p* = 0.038), SD2 0.58 (*p* = 0.029), SD1.5 0.165 (*p* = 0.557), SD1 0.063 (*p* = 0.823)]). Thus, across-trial performance of individual WT mice differed considerably from individual *Fmr1*-KO mice.

### *Fmr1*-KO mice exhibit normal sustained attention and inhibitory control

**Figure 5:**
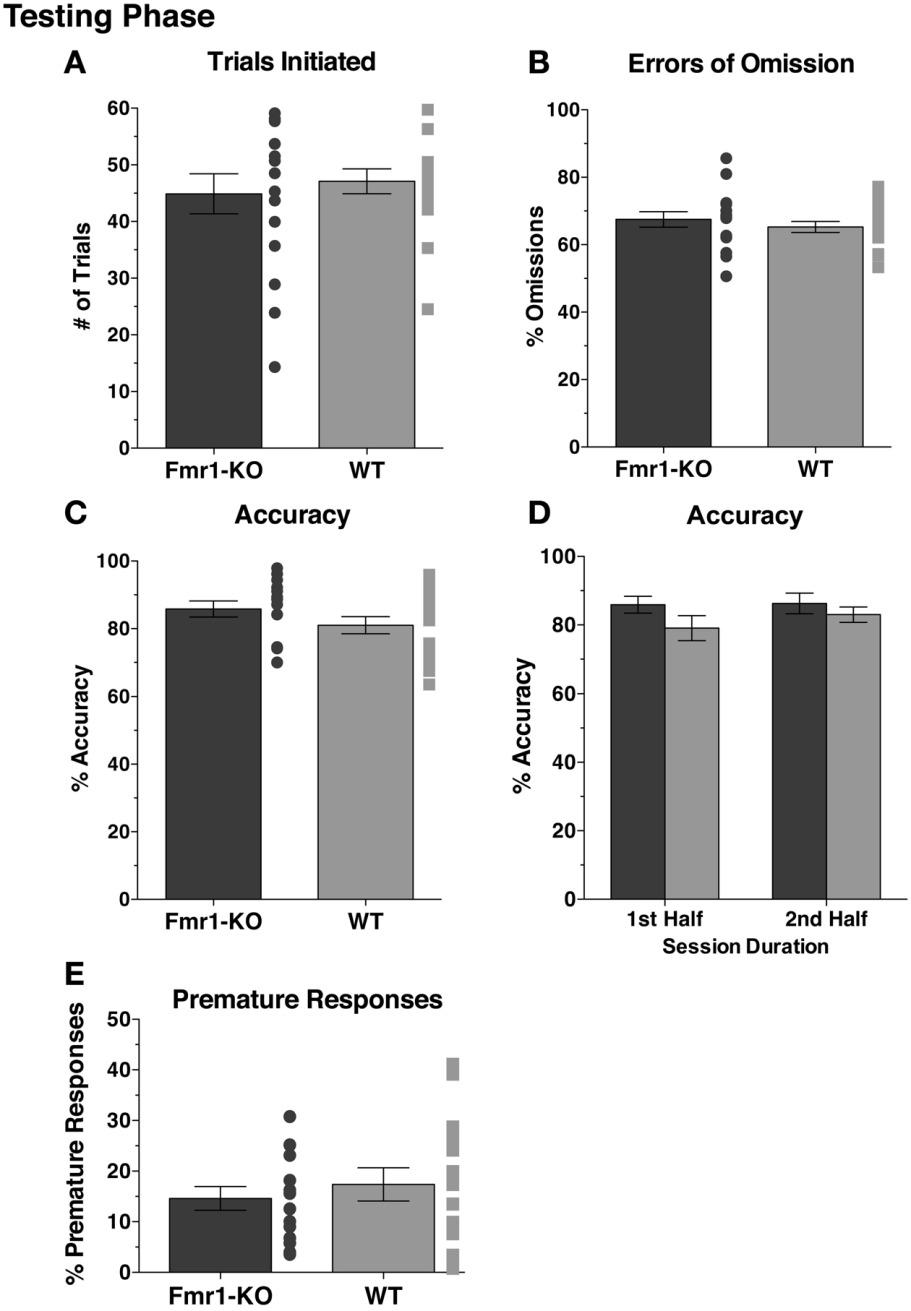
***Fmr1*-KO mice show no deficits in sustained attention and demonstrate no difference in impulsive behaviors during the 5CSRTT SD1 testing phase**. During the 5CSRTT testing phase, with stimulus duration of 1 s (SD1), *Fmr1*-KO and WT mice initiated similar numbers of trials **(A)** and committed equal errors of omission **(B)**. Accuracy for the task was comparable between the two groups **(C)**. Accuracy for either group did not change during the 1st and 2nd half of the session **(D)**. Premature responding **(E)** was equal between *Fmr1*-KO and WT mice.

After 10 sessions of testing at SD1, both groups initiated an equal number of trials (Figure 5A [44.88 ± 3.53 KO, 47.10 ± 2.19 WT] *p* = 0.60). With stimulus duration of 1 s (SD1), a substantial percentage of the trials initiated resulted in omissions, with comparable levels between the two groups (Figure 5B [67.51 ± 2.31 KO, 65.28 ± 1.63 WT] *p* = 0.43). Attention was not impaired in *Fmr1*-KO mice, with accuracy levels matching those of WT mice (Figure 5C [85.82 ± 2.40 KO, 81.01 ± 2.54 WT] *p* = 0.17). Furthermore, attentional performance was maintained throughout session duration for both groups, as indicated by sustained accuracy levels during the 1st and 2nd halves of the testing session (Figure 5D {2-way repeated measurements ANOVA [Session duration effect *F*_(1, 29)_ = 1.16, *p* = 0.30], [Genotype effect *F*_(1, 29)_ = 1.98, *p* = 0.17], [Interaction effect *F*_(1, 29)_ = 0.79, *p* = 0.38]}). Impulsivity, deduced from the amount of premature responses committed, was indistinguishable between the groups (Figure 5E [14.60 ± 2.35 KO, 17.38 ± 3.26 WT] *p* = 0.50).

### MPEP affects performance but not accuracy in the 5CSRTT

**Figure 6:**
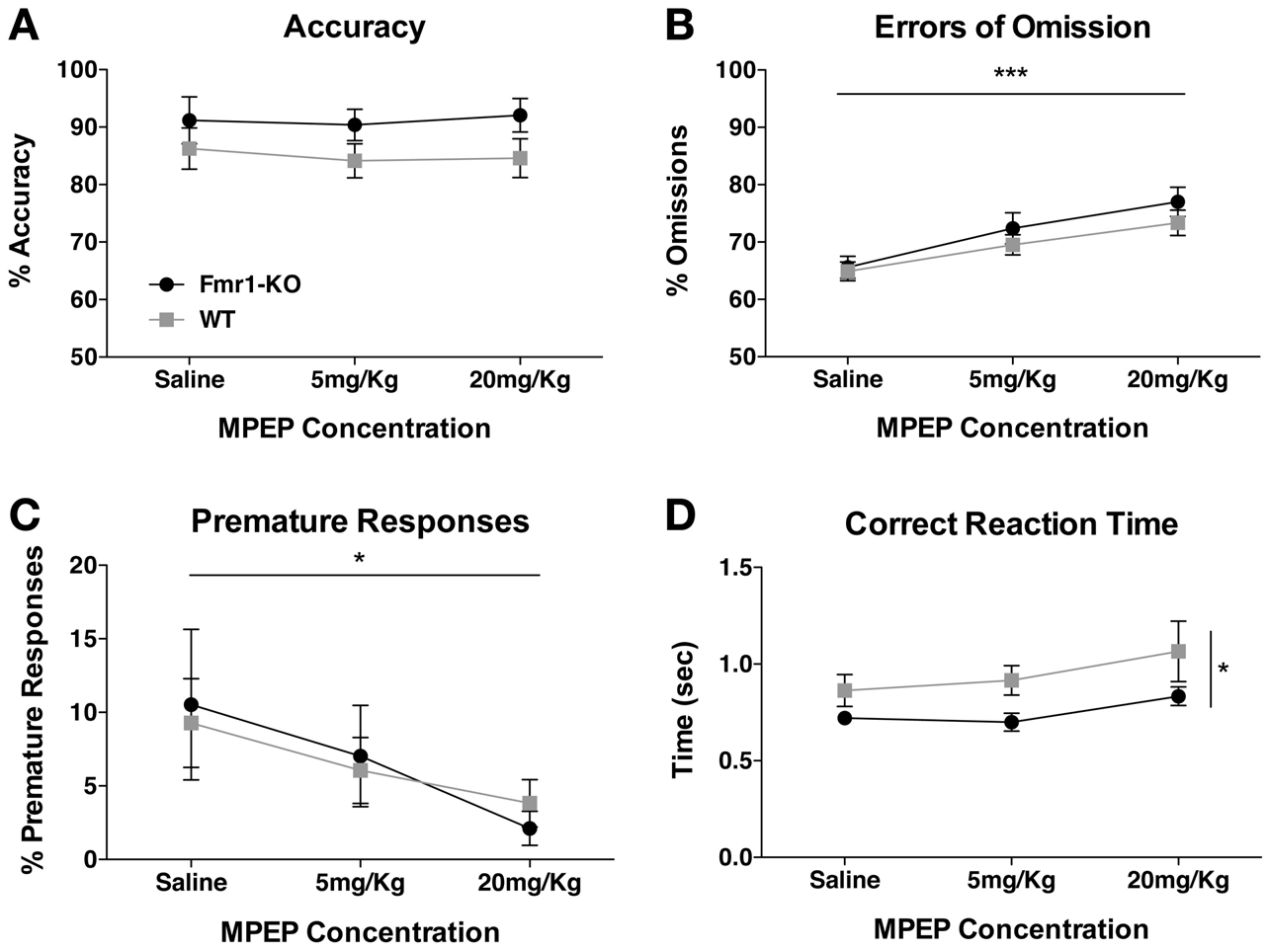
**Effect of MPEP on 5CSRTT performance**. Accuracy in the 5CSRTT testing phase was unaffected by either low (5 mg/Kg) or high (20 mg/Kg) MPEP dosage **(A)**. Errors of omission were significantly enhanced with increasing concentrations of MPEP **(B)**. Premature responses decreased significantly for both groups with increasing MPEP concentrations **(C)**. Reaction time to the correct aperture did not significantly change with MPEP for either group, although *Fmr1*-KO mice reacted faster to the correct aperture **(D)**. Values plotted represent means ± SEM. Analysis was performed with a 2-way repeated measures ANOVA with Bonferroni's post-test analysis. Asterisks indicate significance levels; ^***^p < 0.001, ^*^p < 0.05.

MPEP corrects many behavioral and synaptic phenotypes in the *Fmr1*-KO mouse (Yan et al., 2005; Dolen and Bear, 2008). Following SD1 testing phase, we determined whether MPEP would affect 5CSRTT performance. Acute MPEP administration had no effect upon the number of trials initiated by either group (data not shown, {2-way repeated measurements ANOVA [MPEP effect *F*_(2, 54)_ = 2.43, *p* = 0.10], [Genotype effect *F*_(1, 27)_ = 0.46, *p* = 0.50], [Interaction effect *F*_(2, 54)_ = 0.90, *p* = 0.41]}). Accuracy was also not affected (Figure 6A {2-way repeated measurements ANOVA [MPEP effect *F*_(2, 54)_ = 0.15, *p* = 0.86], [Genotype effect *F*_(1, 27)_ = 3.28, *p* = 0.08], [Interaction effect *F*_(2, 54)_ = 0.11, *p* = 0.90]}), although one animal from each genotype was excluded from analysis due to failure to commit any correct responses after MPEP administration. Errors of omission increased significantly with increasing MPEP concentrations (Figure 6B {2-way repeated measurements ANOVA [MPEP effect *F*_(2, 54)_ = 18.35, *p* < 0.0001], [Genotype effect *F*_(1, 27)_ = 1.06, *p* = 0.31], [Interaction effect *F*_(2, 54)_ = 0.44, *p* = 0.65]}). Impulsive behavior as deduced from premature responding was significantly decreased for both groups with increasing MPEP concentrations (Figure 6C {2-way repeated measurements ANOVA [MPEP effect *F*_(2, 54)_ = 3.29, *p* = 0.04], [Genotype effect *F*_(1, 27)_ = 0.01, *p* = 0.94], [Interaction effect *F*_(2, 54)_ = 0.18, *p* = 0.83]}). Furthermore, reaction time showed a trend for becoming slower with increasing MPEP dosage in both genotypes, with *Fmr1*-KO mice responding overall significantly quicker to the correct aperture, reflective of quicker reaction times during earlier 5CSRTT learning phases (Figure 6D {2-way repeated measurements ANOVA [MPEP effect *F*_(2, 54)_ = 2.26, *p* = 0.10], [Genotype effect *p*_(1, 27)_ = 6.32, *p* = 0.01], [Interaction effect *p*_(2, 54)_ = 0.17, *p* = 0.84]}).

### Increased responding upon rule reversal in *Fmr1*-KO mice

**Figure 7:**
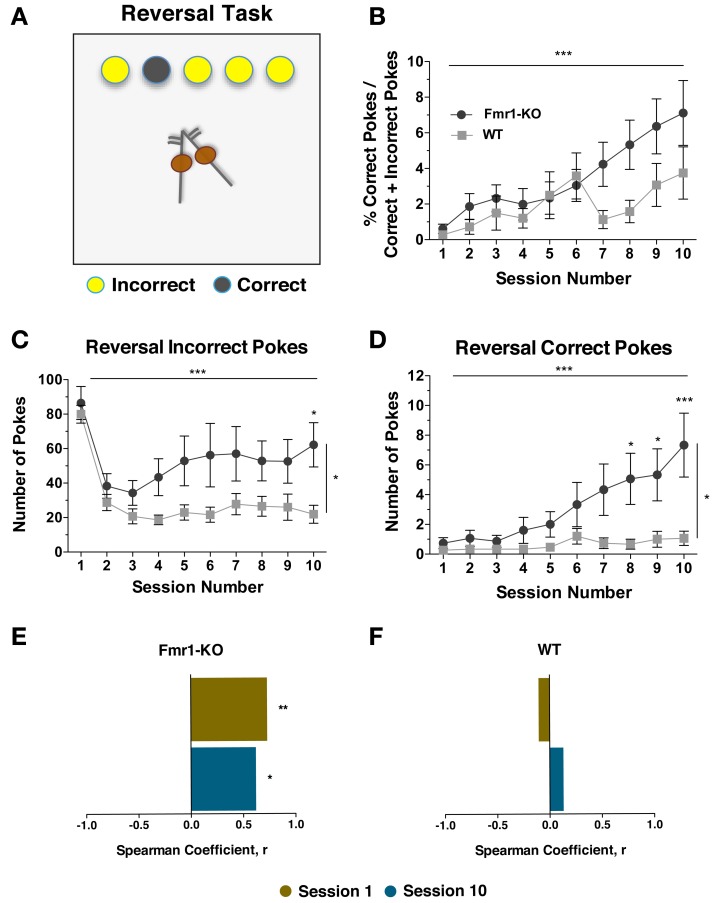
***Fmr1*-KO mice demonstrate enhanced responding upon rule reversal in the 5CSRTT**. During the reversal task a correct trial is now a response in the non-illuminated hole and a response in any of the illuminated holes is deemed incorrect but without consequences **(A)**. After 10 sessions the percentage of correct responses compared to the total responses was not significantly different **(B)**. Both groups of mice performed significantly less incorrect pokes, but *Fmr1*-KO made more incorrect pokes compared to WT mice **(C)**. The number of correct pokes increased significantly for both groups after 10 sessions, with *Fmr1*-KO mice performing more correct responses than WT by the last session **(D)**. Incorrect pokes during L2 correlated with the reversal correct trials for individual *Fmr1*-KO mice during sessions 1 and 10 **(E)**. No such correlation existed for WT mice **(F)**. Values plotted represent means ± SEM. For panels **(B)**, **(C)**, and **(D)** analysis was performed with a 2-way repeated measures ANOVA with Bonferroni's post-test analysis. For all panels asterisks indicate significance levels; ^***^p < 0.001, ^**^p < 0.01, ^*^p < 0.05.

Behavioral inflexibility is a hallmark of ASD (South et al., 2012; D'cruz et al., 2013). To investigate reversal learning, mice were given 10 sessions during which the previously learned association of illuminated response-hole to reward was switched. Under new rules, a reward was now received following a nose-poke in a non-illuminated response-hole. Poking in illuminated response holes was of no consequence (Figure 7A). Both groups initiated similar number of trials although by the last session the trials were equally reduced (data not shown {2-way repeated measurements ANOVA; [Sessions effect *p*_(9, 261)_ = 8.208, *p* < 0.0001], [Genotype effect *p*_(1, 29)_ = 0.700, *p* = 0.411], [Interaction effect *p*_(9, 261)_ = 1.860, *p* = 0.06]}). Compared to the total number of responses, there was a non-significant trend for an increased level of correct poking by *Fmr1*-KO mice by the end of the ten sessions (Figure 7B {2-way repeated measurements ANOVA; [Sessions effect *p*_(9, 261)_ = 5.675, *p* < 0.0001], [Genotype effect *p*_(1, 29)_ = 3.821, *p* = 0.06], [Interaction effect *p*_(9, 261)_ = 1.553, *p* = 0.13]}). However, after ten sessions, *Fmr1*-KO mice continued to make significantly more incorrect pokes compared to WT littermates (Figure 7C {2-way repeated measurements ANOVA; [Sessions effect *p*_(9, 261)_ = 12.30, *p* < 0.0001], [Genotype effect *p*_(1, 29)_ = 5.113, *p* = 0.03], [Interaction effect *p*_(9, 261)_ = 1.543, *p* = 0.13]}). Additionally, by the last session *Fmr1*-KO mice also performed significantly more correct pokes (Figure 7D {2-way repeated measurements ANOVA; [Sessions effect *p*_(9, 261)_ = 7.788, *p* < 0.0001], [Genotype effect *p*_(1, 29)_ = 5.534, *p* = 0.03], [Interaction effect *p*_(9, 261)_ = 4.756, *p* < 0.0001]}). Therefore, although *Fmr1*-KO performance and activity normalized upon completion of the standard 5CSRTT testing, these remerged upon exposure to rule reversal. Interestingly, incorrect poking during L2 significantly correlated with the number of correct trials during the reversal task (in both phases such a response was in the non-illuminated response hole) for individual *Fmr1*-KO mice during sessions 1 and 10 (Figure 7E) whereas no such correlation existed for WT mice (Figure 7F).

## Discussion

FXS is associated with selective impairments in executive function, inhibitory control and specific aspects of attention, which become more pronounced as the cognitive demands of the task increase (Munir et al., 2000; Hagerman, 2006; Scerif et al., 2007; Hooper et al., 2008; Dickson et al., 2013). Given the multicomponent aspects of attentional processing, disrupted in neurodevelopmental disorders (Scerif and Steele, 2011), we assessed attentional processing in *Fmr1*-KO mice using the 5CSRTT. Our results demonstrated an absence of specific impairments in a sustained attentional task in adult male *Fmr1*-KO mice compared with WT littermates. *Fmr1*-KO mice were able to perform similarly to WT mice under increased attentional demand required by short stimulus durations of 1 s, in agreement with a previous study (Krueger et al., 2011). Unlike previous reports using a variation of the 5CSRTT (Moon et al., 2006) we observed no differences in premature responses. Increased arousal arising from the variable cue-onset delay and variable cue duration used in the previous study (Moon et al., 2006) could underlie the reported increase in premature response. In our study, KO mice displayed enhanced responding during the initial 5CSRTT stages of attentional rule acquisition. In addition, KO mice perseveratively poked at the correct aperture during initial training phases but not once the task had been learned. Detailed analysis of task training in each individual mouse demonstrated a significantly altered learning pattern in *Fmr1*-KO mice, with a failure to sustain “correct response” performance and a delay in inhibiting incorrect responses across the training phases. Similar to the initial phases of rule acquisition, *Fmr1*-KO mice also made more responses following rule reversal. Together with the transient hyperactivity observed in two different novel environments, our data suggests that processing of novel stimuli induces heightened activity in *Fmr1*-KO mice that normalizes upon familiarization and habituation to their environment.

### Hyperactivity and increased responding in reaction to novelty

Hyperactivity and attentional impairments are comorbid with a number of psychiatric conditions, including neurodevelopmental disorders (Pliszka, 1998). In *Fmr1*-KO mice, hyperactivity and increased time spent in the center of the OF arena often co-occur, the latter parameter indicating decreased anxiety (Yan et al., 2005; Yuskaitis et al., 2010; Olmos-Serrano et al., 2011; Spencer et al., 2011). We did not observe any difference in levels of anxiety between the two genotypes, as indicated by non-significant differences in overall time spent in the center during both days of testing in the OF arena. However, in our data, distance covered in the OF tests was significantly correlated to the number of center entries made for both genotypes, in line with the idea that general activity and anxiety-related behaviors are linked and cannot easily be dissociated in this assay (Milner and Crabbe, 2008). Hyperactivity is consistently reported in *Fmr1*-KO mice in both young and mature adult stages (Bakker et al., 1994; Mineur et al., 2002; Yuskaitis et al., 2010; Olmos-Serrano et al., 2011; Spencer et al., 2011). Similarly we observed hyperactivity in *Fmr1*-KO mice as compared to WT controls at two different developmental stages in a novel OF arena as well as in a novel home cage. Upon repeated exposure to the OF arena and with subsequent days in the home cage, activity of *Fmr1*-KO mice decreased to WT levels. Thus, in both anxiogenic (OF) and normally reared (home-cage) settings *Fmr1*-KO mice exhibited hyperactivity due to novelty of the environment that normalized with familiarization.

Reflective of the transient hyperactivity observed, was the performance of *Fmr1*-KO mice during acquisition of novel attentional rules and during reversal rule acquisition in the 5CSRTT. Attainment of sustained attentional rules required an activity-dependent progression through the learning and training phases. During both learning phases, KO mice reacted significantly quicker to the light stimulus, initiated more trials, and achieved criterion after fewer sessions, than WT littermates. Furthermore, *Fmr1*-KO mice made significantly more correct and incorrect responses during the second learning phase. This response was not due to motivational differences since the latency to retrieve rewards and the body weight was equal between the two genotypes for both learning sessions. KO mice also made significantly more nose pokes in the magazine during pellet retrieval and were faster in collecting the reward from the magazine than WT littermates. However, these performance measures of correct reaction time, trials initiated, sessions to criterion (Supplementary Figure 2), and number of responses all normalized during training to WT levels. It is therefore evident that novel attentional rules promoted heightened activity and responding in *Fmr1*-KO mice that attenuated with repeated exposure. Furthermore, although activity measures normalized during the 5CSRTT training phase, heightened responding in *Fmr1*-KO mice re-emerged upon rule-reversal.

### Rule reversal

Insistence on sameness and behavioral inflexibility lies at the core of many autistic spectrum disorders (Carcani-Rathwell et al., 2006; Didden et al., 2008). Reversal of a previously-learned rule and adaptation (testing behavioral flexibility in a task) is significantly delayed in children and adolescents with autistic spectrum disorder (South et al., 2012) and impaired in FXS young males (Wilding et al., 2002). Our data show that both groups of mice swiftly (i.e., from the second session onwards) adapted to the new rule by decreasing the number of unrewarded, incorrect responses. WT mice showed less poking overall by the last session, indicative of an extinction response. However, *Fmr1*-KO mice continued to make significantly more errors by continuing to poke illuminated holes and thus were more resistant to adjust to the new rule, or in other words, less able to extinguish responding to the previously rewarded stimulus. KO mice made more correct responses during rule-reversal, but given the concomitant higher level of incorrect responding, there was no significant difference in the proportion of correct responses made overall compared to WT mice. However, with additional training sessions, this trend for increased proportion of correct responses in KO mice could become significant. Furthermore, correct responses during the rule reversal of individual *Fmr1*-KO mice significantly correlated with their incorrect performance during the initial L2 learning phase of 5CSRTT (Figure 7E)—in both cases a poke in a non-illuminated response-hole. Taken together, during rule reversal session, we observed increased levels of both correct and incorrect responding in KO compared to WT mice. Lack of the expected reward upon the performance of a previously learned rule could underlie the increase in arousal and heightened overall responding during rule reversal. This increased activity during acquisition of the novel reverse attentional rules by *Fmr1-*KO mice is also in agreement with previous studies pointing toward increased arousal in *Fmr1*-KO mice upon reversal of a previously learned rule (Moon et al., 2008).

### Training normalizes repetitive behavior in *Fmr1*-KO mice

Repetitive behaviors are a core feature of autistic spectrum disorders (Bodfish et al., 2000). Enhanced repetitive stereotypes are reported in infants with FXS (Baranek et al., 2005) as well as compulsive behaviors in male and female FXS adolescents (Hall et al., 2008). *Fmr1*-KO mice also demonstrated increased repetitive behaviors in the marble burying task (Dansie et al., 2013), in self-grooming (McNaughton et al., 2008), and in stereotypy measures during OF exploration (Hayashi et al., 2007; Dolan et al., 2013). In the 5CSRTT, repetitive behavior is measured as the number of perseverative pokes in the correct illuminated response aperture. We observed enhanced perseverative responses by the *Fmr1*-KO mice during the initial stages of the 5CSRTT training phase compared to WT controls (Figure 3). However, this initial perseveration in *Fmr1*-KO mice normalized with successive training. Young autistic subjects decrease repetitive stereotypic behaviors upon repeated prompting in an attentional task (Chen et al., 2012). It is thus tempting to speculate that repetitive attentional training during the 5CSRTT could underlie the decrease in perseveration observed in *Fmr1*-KO mice in this task.

### Different response performance of individual *Fmr1*-KO mice across 5csrtt

During the training phase, both *Fmr1*-KO and WT groups progressed equally with fewer incorrect responses being made but also fewer correct responses as stimulus duration became shorter and the task became more demanding. However, detailed analysis revealed a significant difference in the performance of individual *Fmr1*-KO mice from one set of trials to the next: individual *Fmr1*-KO mice failed to sustain correct response performance from training to the final testing, SD1 (Figure 4C). KO mice performing the most correct responses at SD16 were amongst the worst performers by SD1, reflected in non-significant correlations for all phases but SD4. In contrast, individual WT mice performed consistently across training phases, with the best performers maintaining the highest number of correct responses throughout all phases (Figure 4B). Our data suggest that even though as a group *Fmr1*-KOs perform equally well as WTs in terms of the number of correct responses, individual *Fmr1*-KO mice lack consistency in correct performance during increasing attentional demands. In contrast to correct responding, individual *Fmr1*-KO mice were more persistent in maintaining incorrect responses for most training phases until SD1.5, indicating a slower change in inhibition of incorrect, unrewarded behaviors (Figure 4F). Conversely, individual WT mice making the highest initial number of incorrect responses at SD16 made the least number of incorrect trials at SD1 (Figure 4E). Thus, although ultimately *Fmr1*-KO and WT as a group reached similar 5CSRTT performance, individual *Fmr1*-KO were slower at inhibiting unrewarded incorrect responses and failed to consistently maintain correct response performance during increasing attentional demands.

### Acute MPEP treatment does not alter 5CSRTT accuracy

Excessive mGluR5 signaling has been widely reported in *Fmr1*-KO mice and it has been shown to underlie several neurophysiological and behavioral deficits observed (Dolen and Bear, 2008; Levenga et al., 2010). Currently phase III clinical trials are underway to test the efficacy of mGluR5 antagonism in treating FXS symptoms. Pharmacological attenuation of mGluR5 signaling with MPEP did not affect 5CSRTT accuracy, but significantly reduced premature responses and increased omission rates in both *Fmr1*-KO and WT mice, mirroring findings in a previous study in rats (Semenova and Markou, 2007). Thus, whilst acute blockade of mGluR5 signaling did cause mice to perform fewer correct trials, it did not cause any adverse effects upon the accuracy of attentional performance, and reduced impulsive behavior. It should be noted here that acute administration of MPEP was conducted in well trained animals when no difference was observed between the two groups. It remains to be investigated whether chronic MPEP administration from the onset of the 5CSRTT can affect the increased responses, heightened activity and perseveration observed in *Fmr1*-KO mice.

Together, these data reveal no impairment in sustained attentional processing in 5CSRTT in the mouse model for FXS. The lack of a sustained attentional deficit but subtle differences in learning raise the possibilities that in the mouse, the *Fmr1* gene is not necessary for sustained attention or that compensatory changes occur as training progresses so that any initial learning impairments arising from lack of FMRP are overcome (Crawley, 2000). FMRP is lacking from the entire brain thus it is difficult to pinpoint where any potential compensation may occur, although similarities of altered cerebellar- PFC circuit function are reported for *Fmr1*-KO and cerebellar-specific mutants with autistic behavioral phenotype (Rogers et al., 2013). However, our analysis reveals that *Fmr1*-KO mice respond differently when presented with novel rules or new environments. During acquisition of novel attentional rules *Fmr1*-KO mice display increased activity, heightened response levels, and perseveration that normalizes with repeated training. Similarly, exposure to novel environments induces hyperactivity in *Fmr1*-KO mice that subsides with familiarization. Additionally, we demonstrate that individual KO mice fail to perform consistently during the 5CSRTT training phase and are delayed in inhibiting incorrect responses. Finally, we demonstrate for the first time that acute mGluR5 blockade, whilst increasing omitted trials and blocking impulsive responses, does not impair accuracy in the *Fmr1*-KO mouse model.

## Author contributions

Ioannis Kramvis collected all behavioral data from WT and Fmr1-KO mice in this study. Ioannis Kramvis analyzed all data, using tools developed by Maarten Loos. Maarten Loos collected and analyzed the novel home-cage data. All authors designed the experiments and Ioannis Kramvis, Maarten Loos, Rhiannon Meredith wrote the manuscript.

## Conflict of interest statement

The authors declare that the research was conducted in the absence of any commercial or financial relationships that could be construed as a potential conflict of interest.

## Supplementary material

Supplementary Figure 1 **Increased velocity during introduction to the novel open field arena by *Fmr1*-KO mice**. The degree of mobility for either young adult **(A)** or mature adult **(B)** mice is comparable between the two groups during both days of novel open field exploration and decreases significantly upon re-exposure to the arena. Young adult *Fmr1*-KO mice are significantly faster than WT controls during the first but not the second day of novel open field exploration **(C)**. During the first but not the second day of novel open field exploration mature adult *Fmr1*-KO mice also move significantly faster that WT controls **(D)**. Values plotted represent means ± SEM. Analysis was performed with a 2-way repeated measures ANOVA with Bonferroni's post-test analysis. Asterisks indicate significance levels; ^***^*p* < 0.001, ^*^*p* < 0.05.

Supplementary Figure 2 ***Fmr1*-KO mice performance and motivation is comparable to WT throughout the 5CSRTT training phase**. The number of sessions to achieve criterion increases significantly for both groups with progressive shorter stimulus duration **(A)**. *Fmr1*-KO and WT mice initiate equal number of trials during training stage of the 5CSRTT **(B)**. With training and progressively shorter stimulus duration reaction time to the correct aperture decreases significantly for both groups **(C)**. Latency to retrieve the magazine reward remains constant through the training stages of the 5CSRTT **(D)**. Values plotted represent means ± SEM. Analysis was performed with a 2-way repeated measures ANOVA with Bonferroni's post-test analysis. Asterisks indicate significance levels; ^***^*p* < 0.001.

## References

[B1] Bakker C. E. Verheij C. Willemsen R. van der Helm R. Oerlemans F. Vermey M. (1994). Fmr1 knockout mice: a model to study fragile X mental retardation. The Dutch-Belgian Fragile X Consortium. Cell 78, 23–33 8033209

[B2] Baranek G. T. Danko C. D. Skinner M. L. Bailey D. B. Jr. Hatton D. D. Roberts J. E. (2005). Video analysis of sensory-motor features in infants with fragile X syndrome at 9-12 months of age. J. Autism Dev. Disord. 35, 645–656 10.1007/s10803-005-0008-7 16172809

[B3] Bodfish J. W. Symons F. J. Parker D. E. Lewis M. H. (2000). Varieties of repetitive behavior in autism: comparisons to mental retardation. J. Autism Dev. Disord. 30, 237–243 10.1023/A:1005596502855 11055459

[B4] Carcani-Rathwell I. Rabe-Hasketh S. Santosh P. J. (2006). Repetitive and stereotyped behaviours in pervasive developmental disorders. J. Child Psychol. Psychiatry 47, 573–581 10.1111/j.1469-7610.2005.01565.x 16712634

[B5] Chen G. M. Yoder K. J. Ganzel B. L. Goodwin M. S. Belmonte M. K. (2012). Harnessing repetitive behaviours to engage attention and learning in a novel therapy for autism: an exploratory analysis. Front. Psychol. 3:12 10.3389/fpsyg.2012.00012 22355292 PMC3280620

[B6] Cornish K. Munir F. Wilding J. (2001). [A neuropsychological and behavioural profile of attention deficits in fragile X syndrome]. Rev. Neurol. 33(Suppl. 1), S24–S29 12447815

[B7] Crawley J. (ed.). (2007). What's Wrong With My Mouse? Behavioral Phenotyping of Transgenic and Knockout Mice. (New York, NY: Wiley-Liss). 10.1002/0470119055

[B8] Crawley J. N. (2000). Behavioral phenotyping of transgenic and knockout mice: experimental design and evaluation of general health, sensory functions, motor abilities, and specific behavioral tests. ILAR J. 41, 136–143 10.1093/ilar.41.3.136 10448192

[B9] Dalley J. W. Cardinal R. N. Robbins T. W. (2004). Prefrontal executive and cognitive functions in rodents: neural and neurochemical substrates. Neurosci. Biobehav. Rev. 28, 771–784 10.1016/j.neubiorev.2004.09.006 15555683

[B10] Dansie L. E. Phommahaxay K. Okusanya A. G. Uwadia J. Huang M. Rotschafer S. E. (2013). Long-lasting effects of minocycline on behavior in young but not adult Fragile X mice. Neuroscience 246, 186–198 10.1016/j.neuroscience.2013.04.058 23660195 PMC3813005

[B11] D'cruz A. M. Ragozzino M. E. Mosconi M. W. Shrestha S. Cook E. H. Sweeney J. A. (2013). Reduced behavioral flexibility in autism spectrum disorders. Neuropsychology 27, 152–160 10.1037/a0031721 23527643 PMC3740947

[B12] Dickson P. E. Corkill B. McKimm E. Miller M. M. Calton M. A. Goldowitz D. (2013). Effects of stimulus salience on touchscreen serial reversal learning in a mouse model of fragile X syndrome. Behav. Brain Res. 252, 126–135 10.1016/j.bbr.2013.05.060 23747611 PMC3854797

[B13] Didden R. Sigafoos J. Green V. A. Korzilius H. Mouws C. Lancioni G. E. (2008). Behavioural flexibility in individuals with Angelman syndrome, Down syndrome, non-specific intellectual disability and Autism spectrum disorder. J. Intellect. Disabil. Res. 52, 503–509 10.1111/j.1365-2788.2008.01055.x 18384537

[B14] Dolan B. M. Duron S. G. Campbell D. A. Vollrath B. Shankaranarayana Rao B. S. Ko H. Y. (2013). Rescue of fragile X syndrome phenotypes in Fmr1 KO mice by the small-molecule PAK inhibitor FRAX486. Proc. Natl. Acad. Sci. U.S.A. 110, 5671–5676 10.1073/pnas.1219383110 23509247 PMC3619302

[B15] Dolen G. Bear M. F. (2008). Role for metabotropic glutamate receptor 5 (mGluR5) in the pathogenesis of fragile X syndrome. J. Physiol. 586, 1503–1508 10.1113/jphysiol.2008.150722 18202092 PMC2375688

[B16] Gocel J. Larson J. (2012). Synaptic NMDA receptor-mediated currents in anterior piriform cortex are reduced in the adult fragile X mouse. Neuroscience 221, 170–181 10.1016/j.neuroscience.2012.06.052 22750206 PMC3424403

[B17] Hagerman R. J. M. D. (2006). Lessons from Fragile X regarding neurobiology, autism, and neurodegeneration. J. Dev. Behav. Pediatr. 27, 63–74 10.1097/00004703-200602000-00012 16511373

[B18] Hall S. S. Lightbody A. A. Reiss A. L. (2008). Compulsive, self-injurious, and autistic behavior in children and adolescents with fragile X syndrome. Am. J. Ment. Retard. 113, 44–53 10.1352/0895-8017(2008)113[44:CSAABI]2.0.CO;2 18173299

[B19] Hayashi M. L. Rao B. S. Seo J. S. Choi H. S. Dolan B. M. Choi S. Y. (2007). Inhibition of p21-activated kinase rescues symptoms of fragile X syndrome in mice. Proc. Natl. Acad. Sci. U.S.A. 104, 11489–11494 10.1073/pnas.0705003104 17592139 PMC1899186

[B20] Hooper S. R. Hatton D. Sideris J. Sullivan K. Hammer J. Schaaf J. (2008). Executive functions in young males with fragile X syndrome in comparison to mental age-matched controls: baseline findings from a longitudinal study. Neuropsychology 22, 36–47 10.1037/0894-4105.22.1.36 18211154

[B21] Knudsen E. I. (2007). Fundamental components of attention. Annu. Rev. Neurosci. 30, 57–78 10.1146/annurev.neuro.30.051606.094256 17417935

[B22] Krueger D. D. Osterweil E. K. Chen S. P. Tye L. D. Bear M. F. (2011). Cognitive dysfunction and prefrontal synaptic abnormalities in a mouse model of fragile X syndrome. Proc. Natl. Acad. Sci. U.S.A. 108, 2587–2592 10.1073/pnas.1013855108 21262808 PMC3038768

[B23] Levenga J. De Vrij F. M. Oostra B. A. Willemsen R. (2010). Potential therapeutic interventions for fragile X syndrome. Trends Mol. Med. 16, 516–527 10.1016/j.molmed.2010.08.005 20864408 PMC2981507

[B24] Loos M. Van Der Sluis S. Bochdanovits Z. Van Zutphen I. J. Pattij T. Stiedl O. (2009). Activity and impulsive action are controlled by different genetic and environmental factors. Genes Brain Behav. 8, 817–828 10.1111/j.1601-183X.2009.00528.x 19751396

[B25] Maroteaux G. Loos M. Van Der Sluis S. Koopmans B. Aarts E. Van Gassen K. (2012). High-throughput phenotyping of avoidance learning in mice discriminates different genotypes and identifies a novel gene. Genes Brain Behav. 11, 772–784 10.1111/j.1601-183X.2012.00820.x 22846151 PMC3508728

[B26] McNaughton C. H. Moon J. Strawderman M. S. Maclean K. N. Evans J. Strupp B. J. (2008). Evidence for social anxiety and impaired social cognition in a mouse model of fragile X syndrome. Behav. Neurosci. 122, 293–300 10.1037/0735-7044.122.2.293 18410169

[B27] Meredith R. M. Holmgren C. D. Weidum M. Burnashev N. Mansvelder H. D. (2007). Increased threshold for spike-timing-dependent plasticity is caused by unreliable calcium signaling in mice lacking fragile X gene FMR1. Neuron 54, 627–638 10.1016/j.neuron.2007.04.028 17521574

[B28] Milner L. C. Crabbe J. C. (2008). Three murine anxiety models: results from multiple inbred strain comparisons. Genes Brain Behav. 7, 496–505 10.1111/j.1601-183X.2007.00385.x 18182070

[B29] Mineur Y. S. Sluyter F. De Wit S. Oostra B. A. Crusio W. E. (2002). Behavioral and neuroanatomical characterization of the Fmr1 knockout mouse. Hippocampus 12, 39–46 10.1002/hipo.10005 11918286

[B30] Moon J. Beaudin A. E. Verosky S. Driscoll L. L. Weiskopf M. Levitsky D. A. (2006). Attentional dysfunction, impulsivity, and resistance to change in a mouse model of fragile X syndrome. Behav. Neurosci. 120, 1367–1379 10.1037/0735-7044.120.6.1367 17201482

[B31] Moon J. Ota K. T. Driscoll L. L. Levitsky D. A. Strupp B. J. (2008). A mouse model of fragile X syndrome exhibits heightened arousal and/or emotion following errors or reversal of contingencies. Dev. Psychobiol. 50, 473–485 10.1002/dev.20308 18551464

[B32] Munir F. Cornish K. M. Wilding J. (2000). A neuropsychological profile of attention deficits in young males with fragile X syndrome. Neuropsychologia 38, 1261–1270 10.1016/S0028-3932(00)00036-1 10865102

[B33] Olmos-Serrano J. L. Corbin J. G. Burns M. P. (2011). The GABA(A) receptor agonist THIP ameliorates specific behavioral deficits in the mouse model of fragile X syndrome. Dev. Neurosci. 33, 395–403 10.1159/000332884 22067669 PMC3254038

[B34] Paul K. Venkitaramani D. V. Cox C. L. (2013). Dampened dopamine-mediated neuromodulation in prefrontal cortex of fragile X mice. J. Physiol. 591, 1133–1143 10.1113/jphysiol.2012.241067 23148316 PMC3591719

[B35] Peyrache A. Khamassi M. Benchenane K. Wiener S. I. Battaglia F. P. (2009). Replay of rule-learning related neural patterns in the prefrontal cortex during sleep. Nat. Neurosci. 12, 919–926 10.1038/nn.2337 19483687

[B36] Pliszka S. R. (1998). Comorbidity of attention-deficit/hyperactivity disorder with psychiatric disorder: an overview. J. Clin. Psychiatry 59(Suppl. 7), 50–58 9680053

[B37] Robbins T. W. (2002). The 5-choice serial reaction time task: behavioural pharmacology and functional neurochemistry. Psychopharmacology (Berl.) 163, 362–380 10.1007/s00213-002-1154-7 12373437

[B38] Rogers T. D. Dickson P. E. McKimm E. Heck D. H. Goldowitz D. Blaha C. D. (2013). Reorganization of circuits underlying cerebellar modulation of prefrontal cortical dopamine in mouse models of autism spectrum disorder. Cerebellum 12, 547–556 10.1007/s12311-013-0462-2 23436049 PMC3854915

[B39] Rossi A. F. Pessoa L. Desimone R. Ungerleider L. G. (2009). The prefrontal cortex and the executive control of attention. Exp. Brain Res. 192, 489–497 10.1007/s00221-008-1642-z 19030851 PMC2752881

[B40] Scerif G. Cornish K. Wilding J. Driver J. Karmiloff-Smith A. (2007). Delineation of early attentional control difficulties in fragile X syndrome: focus on neurocomputational changes. Neuropsychologia 45, 1889–1898 10.1016/j.neuropsychologia.2006.12.005 17254617 PMC2613507

[B41] Scerif G. Steele A. (2011). Neurocognitive development of attention across genetic syndromes: inspecting a disorder's dynamics through the lens of another. Prog. Brain Res. 189, 285–301 10.1016/B978-0-444-53884-0.00030-0 21489395

[B42] Semenova S. Markou A. (2007). The effects of the mGluR5 antagonist MPEP and the mGluR2/3 antagonist LY341495 on rats' performance in the 5-choice serial reaction time task. Neuropharmacology 52, 863–872 10.1016/j.neuropharm.2006.10.003 17126859 PMC1847349

[B43] South M. Newton T. Chamberlain P. D. (2012). Delayed reversal learning and association with repetitive behavior in autism spectrum disorders. Autism Res. 5, 398–406 10.1002/aur.1255 23097376

[B44] Spencer C. M. Alekseyenko O. Hamilton S. M. Thomas A. M. Serysheva E. Yuva-Paylor L. A. (2011). Modifying behavioral phenotypes in Fmr1KO mice: genetic background differences reveal autistic-like responses. Autism Res. 4, 40–56 10.1002/aur.168 21268289 PMC3059810

[B45] Testa-Silva G. Loebel A. Giugliano M. De Kock C. P. Mansvelder H. D. Meredith R. M. (2012). Hyperconnectivity and slow synapses during early development of medial prefrontal cortex in a mouse model for mental retardation and autism. Cereb. Cortex 22, 1333–1342 10.1093/cercor/bhr224 21856714 PMC3561643

[B46] Wilding J. Cornish K. Munir F. (2002). Further delineation of the executive deficit in males with fragile-X syndrome. Neuropsychologia 40, 1343–1349 10.1016/S0028-3932(01)00212-3 11931937

[B47] Yan Q. J. Rammal M. Tranfaglia M. Bauchwitz R. P. (2005). Suppression of two major Fragile X Syndrome mouse model phenotypes by the mGluR5 antagonist MPEP. Neuropharmacology 49, 1053–1066 10.1016/j.neuropharm.2005.06.004 16054174

[B48] Yuskaitis C. J. Mines M. A. King M. K. Sweatt J. D. Miller C. A. Jope R. S. (2010). Lithium ameliorates altered glycogen synthase kinase-3 and behavior in a mouse model of fragile X syndrome. Biochem. Pharmacol. 79, 632–646 10.1016/j.bcp.2009.09.023 19799873 PMC2810609

