# Supplementary figures and images for: Hyperactivity, perseveration and increased responding during attentional rule acquisition in the Fragile X mouse model
Source: Front Behav Neurosci. 2013 Nov 21;7:172. doi: 10.3389/fnbeh.2013.00172 (PMC3836024; doi:10.3389/fnbeh.2013.00172)

**(a) Activity Young Adult**

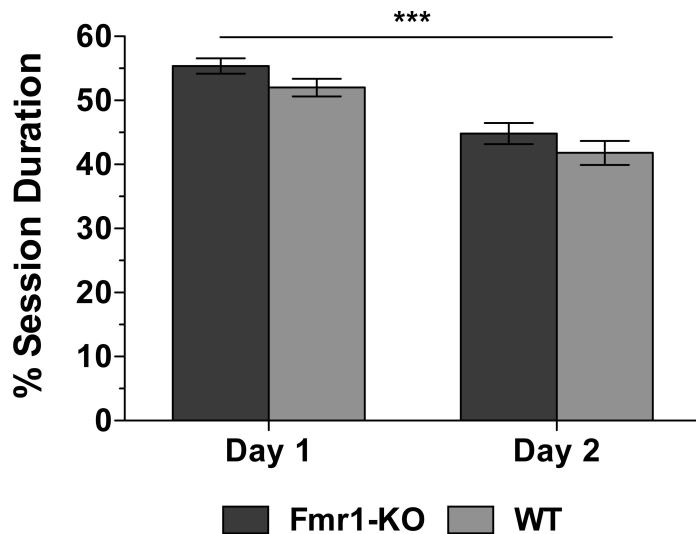

**(b) Activity Mature Adult**

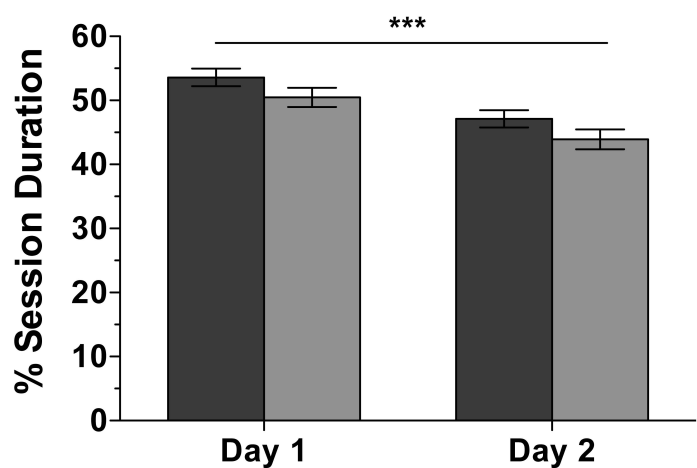

**(c) Velocity Young Adult**

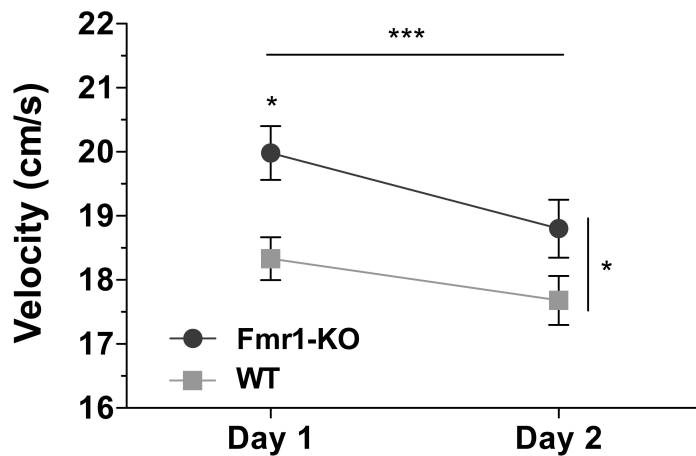

**(d) Velocity Mature Adult**

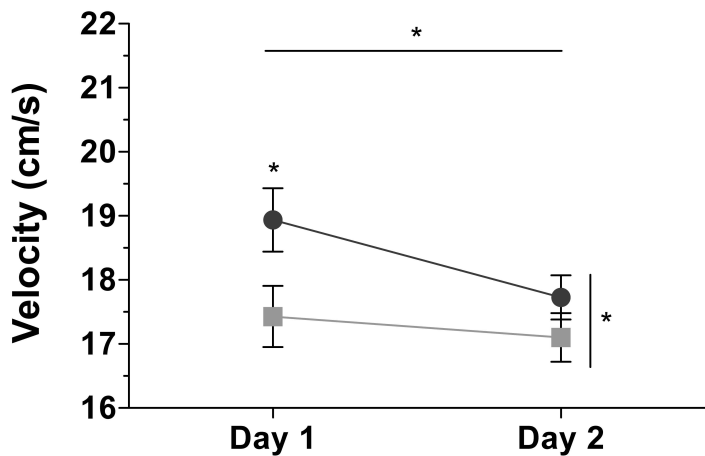

Supplement: Supplementary Figure 1 — Increased velocity during introduction to the novel open field arena by Fmr1-KO mice. The degree of mobility for either young adult (A) or mature adult (B) mice is comparable between the two groups during both days of novel open field exploration and decreases significantly upon re-exposure to the arena. Young adult Fmr1-KO mice are significantly faster than WT controls during the first but not the second day of novel open field exploration (C). During the first but not the second day of novel open field exploration mature adult Fmr1-KO mice also move significantly faster that WT controls (D). Values plotted represent means ± SEM. Analysis was performed with a 2-way repeated measures ANOVA with Bonferroni's post-test analysis. Asterisks indicate significance levels; ***p < 0.001, *p < 0.05. [file DataSheet3.PDF]

**(a)****Sessions To Criterion**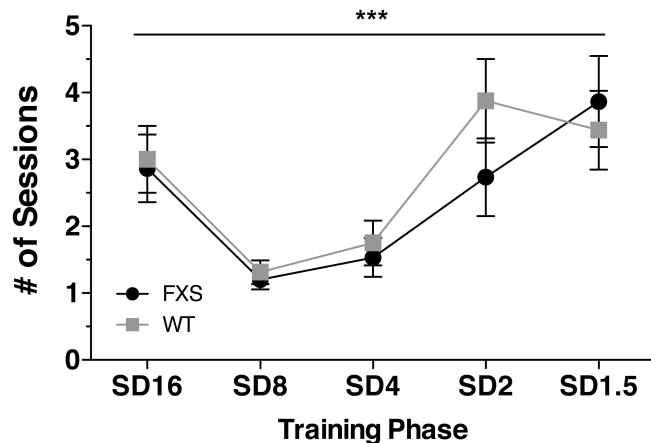**(b)****Trials Initiated**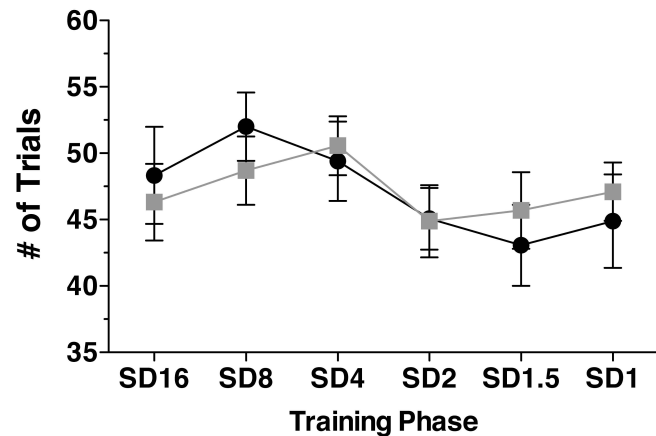**(c)****Correct Reaction Time**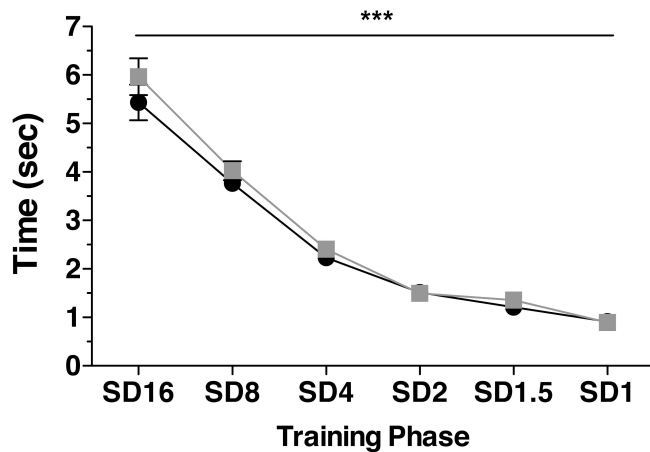**(d)****Reward Latency**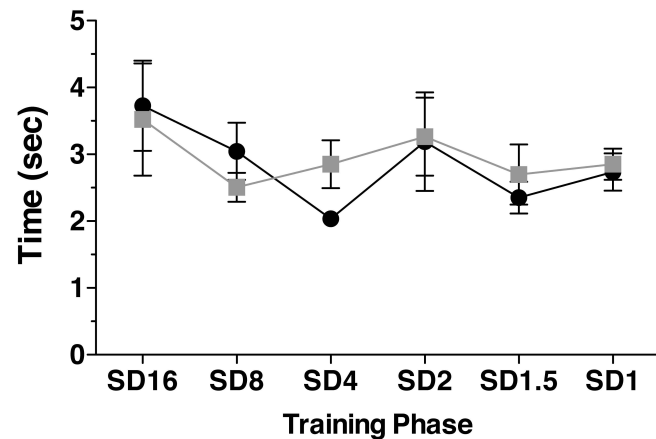

Supplement: Supplementary Figure 2 — Fmr1-KO mice performance and motivation is comparable to WT throughout the 5CSRTT training phase. The number of sessions to achieve criterion increases significantly for both groups with progressive shorter stimulus duration (A). Fmr1-KO and WT mice initiate equal number of trials during training stage of the 5CSRTT (B). With training and progressively shorter stimulus duration reaction time to the correct aperture decreases significantly for both groups (C). Latency to retrieve the magazine reward remains constant through the training stages of the 5CSRTT (D). Values plotted represent means ± SEM. Analysis was performed with a 2-way repeated measures ANOVA with Bonferroni's post-test analysis. Asterisks indicate significance levels; ***p < 0.001. [file DataSheet4.PDF]
